# Use of Propylene-Glycol as a Cosolvent for GnRH in Synchronization of Estrus and Ovulation in Sheep

**DOI:** 10.3390/ani10050897

**Published:** 2020-05-21

**Authors:** Zurisaday Santos-Jimenez, Sara Guillen-Gargallo, Teresa Encinas, Fiammetta Berlinguer, Francisco G. Veliz-Deras, Paula Martinez-Ros, Antonio Gonzalez-Bulnes

**Affiliations:** 1 Departamento de Farmacologia y Toxicologia Facultad de Veterinaria, UCM, Ciudad Universitaria s/n, 28040 Madrid, Spain; mvz_zusan@hotmail.com (Z.S.-J.); tencinas@ucm.es (T.E.); bulnes@inia.es (A.G.-B.); 2 Universidad Autónoma Agraria Antonio Narro, Torreón, Coahuila 25315, Mexico; velizderas@gmail.com; 3 Departamento de Produccion y Sanidad Animal, Facultad de Veterinaria, Universidad Cardenal Herrera-CEU, CEU Universities, C/ Tirant lo Blanc, 7, Alfara del Patriarca, 46115 Valencia, Spain; guigarsar@alumnos.uchceu.es; 4 Dipartamento di Medicina Veterinaria, Università degli Studi di Sassari, Via Vienna 2, 07100 Sassari, Italy; berling@uniss.it; 5 Departamento de Reproduccion Animal, INIA, Avda. Puerta de Hierro s/n, 28040 Madrid, Spain

**Keywords:** eCG, estrus synchronization, GnRH, propylene-glycol, sheep

## Abstract

**Simple Summary:**

The foreseeable shortage of equine chorionic gonadotrophin (eCG) for estrus synchronization in sheep will have a huge impact on breeders using out-of-season breeding, artificial insemination, or embryo transfer because there is no other product with similar activity. Hence, there is a strong need for alternative protocols. The aim of the present work was to evaluate the reproductive response of sheep in breeding season to the administration of gonadotrophin-releasing hormone (GnRH) using propylene-glycol as a cosolvent, as well as the most adequate timing for such administration. From the results obtained, protocols based on a short-term treatment with a Controlled Internal Drug Release (CIDR) device and a single dose of GnRH in propylene-glycol at 36 h after CIDR removal may constitute an alternative to traditional protocols based on the administration of a single dose of eCG at CIDR removal.

**Abstract:**

The foreseen shortage of eCG for estrus synchronization in sheep makes necessary the development of alternative protocols. The aim of the present work was to evaluate the reproductive response of sheep in breeding season to the administration of GnRH using propylene-glycol as a cosolvent and the subcutaneous route for slowing and extending the release of GnRH, as well as the most adequate timing for such administration. In the present study, protocols based on a short-term CIDR treatment and a single subcutaneous dose of GnRH in propylene-glycol at 36 h after CIDR removal induced a similar ovarian response to protocols based on administration of eCG at CIDR removal or intramuscular GnRH in distilled water at 56 h after. In such protocol, 80% of the animals developed estrus in a narrow timing (75% between 36 and 48 h after CIDR removal), and all of them also ovulated in a narrow window (87.5% between 72 and 76 h after CIDR removal, with 62.5% between 72 and 76 h) and showed a similar ovulation rate and plasma progesterone concentrations at the induced estrous cycle. Hence, administration of GnRH in propylene-glycol may constitute an alternative to traditional protocols based on the administration of eCG.

## 1. Introduction

Induction and synchronization of estrus and ovulation in sheep, in both breeding or non-breeding season and both for natural mating or artificial insemination, mostly rely on the use of protocols including the insertion of intravaginal devices with progesterone or progestagens and a single dose of equine chorionic gonadotrophin (eCG) at device removal [1]. The administration of eCG was found, in the early 1950s, to be successful for inducing estrus and ovulation during seasonal anoestrus [2,3]. Afterwards, was eCG found to also be effective for ensuring a narrow window of ovulation among ewes in the same lot and therefore was incorporated into first protocols including fixed-time artificial insemination (FTAI), which was developed in the 1960s [4,5]. These protocols have been carried on for decades, with changes only having been made in the type and duration of the progestative treatments, but always maintaining eCG as an essential component.

The future availability and use of eCG, however, is currently compromised because it is obtained by bleeding pregnant mares, and the societal pressure against companies manufacturing the hormone may force them to discontinue its production [6]. The banning of eCG will have a huge impact on sheep producers using out-of-season breeding, artificial insemination, or embryo transfer because there is no other product with similar activity [7]. Hence, there is a strong need for alternative protocols.

Our group has developed a protocol using gonadotrophin-releasing hormone (GnRH) instead of eCG [7]. Such protocol has proved to be useful in breeding season, with a percentage of sheep responding with estrus and ovulation similar to eCG-based treatments (around 85–95%), but unfeasible during non-breeding season, with only 35–48% of ewes showing estrus behavior (unpublished results). However, almost all (83–96%) of the treated sheep were found to be ovulated in spite of the absence of estrus signs. Concomitantly, occurrence of estrus was also slightly affected during breeding season [8]; its onset occurred later in GnRH-treated sheep compared with eCG-treated sheep, although intervals among timing of estrus-preovulatory luteinizing hormone (LH) peak-ovulation were similar with both hormones. Our hypothesis for such absence or delay of estrus signs would be that a single GnRH injection could not stimulate an adequate development and maturation of the preovulatory follicle. Available evidence indicates that the administration of GnRH induces a sharp discharge of LH, which in turns induces ovulation [9], without the sustained follicle stimulating hormone (FSH) and LH activity provided by the long half-life of eCG. Half-life of GnRH is indeed 2–4 min because it is rapidly degraded by peptidases and cleared by glomerular filtration [10]. Hence, in GnRH-treated sheep, there would be a lack of the LH pulsatility that is necessary for final follicle maturation and onset of estrus behavior [11].

Our second hypothesis is that occurrence and features of estrus and ovulation would be improved by slowing and extending the release of GnRH. Such an effect may be accomplished by inducing a prolonged absorption phase with a solvent, allowing a long-acting formulation [12]. The choice should only consider those solvents that are generally recognized as safe (GRAS) [13]. There are different nonionic surfactants considered GRAS, such as polyethylene-glycol and propylene-glycol, which provide slower drug absorption from the injection site and higher bioavailability [14]. Propylene-glycol, when used as a cosolvent for peptide hormones, has even shown to induce higher bioavailability than polyethylene-glycol (around 60% and 12% higher than water solutions, respectively) [15]. Moreover, propylene-glycol has low systemic toxicity, and exposure of laboratory animals has only been associated with reversible hematological changes [16]; the same has been found in sheep (unpublished results). The compound has been successfully used as a cosolvent for long-acting parenteral administration of both peptide and steroid hormones in sheep [17,18], with the subcutaneous route inducing both longer mean residence time and longer above-threshold value time of the hormone than the intramuscular route [18]. Furthermore, the use of subcutaneous administration induces fewer problems of precipitation, pain, inflammation, and hemolysis than the intramuscular injection [19,20].

Hence, prior to further studies in non-breeding season and FTAI protocols, the aims of the present work were to evaluate (a) the reproductive response of sheep in breeding season to the administration of GnRH using propylene-glycol as a cosolvent, and (b) the most adequate timing for such administration. Two consecutive experiments were performed, the first trial used a prostaglandin-based protocol to determine the effect of including GnRH in either distilled water or propylene glycol on the ovarian response and, subsequently, the second trial aimed to determine the efficiency of including GnRH in propylene-glycol in progesterone-based protocols.

## 2. Materials and Methods

### 2.1. Animals and Ethical Issues

The study included two consecutive trials that were carried out during the breeding season (end of October and early November), involving 55 Segureña sheep (2–5 years-old, with a mean body condition of 3.5 ± 0.5) at the experimental flock of the CEU Cardenal Herrera University in Naquera (Valencia, Spain; latitude 39 °N). The experiments were performed according to the Spanish Policy for Animal Protection RD53/2013, which meets the European Union Directive 2010/63/UE about the protection of animals used for research, and was specifically assessed and approved by the CEU Cardenal Herrera Committee of Ethics in Animal Research (report CEEA17/019).

### 2.2. Experimental Design

**Figure 1:**
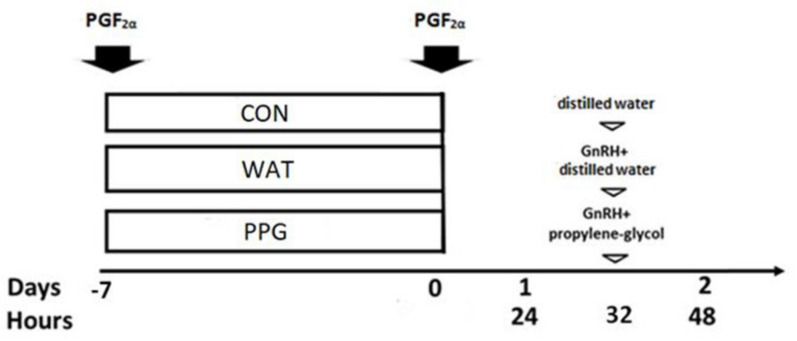
Experimental groups of trial 1, aiming to determine the effect of gonadotrophin-releasing hormone (GnRH) administration in either distilled water or propylene-glycol on the ovarian response to prostaglandin-based protocols. All females were treated with two doses of prostaglandin F_2α_ (PGF_2α_) 7 days apart and, 32 h after injecting the second dose, they received either 5 mL of distilled water (group CON, *n* = 6) or 1 mL of Acegon (50 μg of GnRH) in either i.m. distilled water (group WAT, *n* = 6) or i.m. 4 mL of propylene-glycol (group PPG, *n* = 6).

The first trial aimed at determining the effect of GnRH administration in either distilled water or propylene-glycol on the ovarian response to prostaglandin-based protocols for estrus synchronization. Ovarian cyclic activity and ovulation were synchronized, in a total of 18 ewes, by the i.m. administration of two doses of prostaglandin F_2α_ (5 mg of Dinolytic (Zoetis, Madrid, Spain)), 7 days apart. At 32 h after the second prostaglandin dose, a third of the animals received one s.c. injection of 5 mL of distilled water and remained as controls (group CON, *n* = 6), whereas the remaining ewes were injected with 50 μg of GnRH (Acegon, Lab. Syva, Leon, Spain), in either a single i.m. dose in distilled water (group WAT, *n* = 6) or a single s.c. dose in propylene-glycol (group PPG, *n* = 6), as depicted in Figure 1. The GnRH solution in the group PPG was prepared by mixing 1 mL of Acegon (equivalent to 50 μg of GnRH) with 4 mL of propylene-glycol, as previously described [16,17].

**Figure 2:**
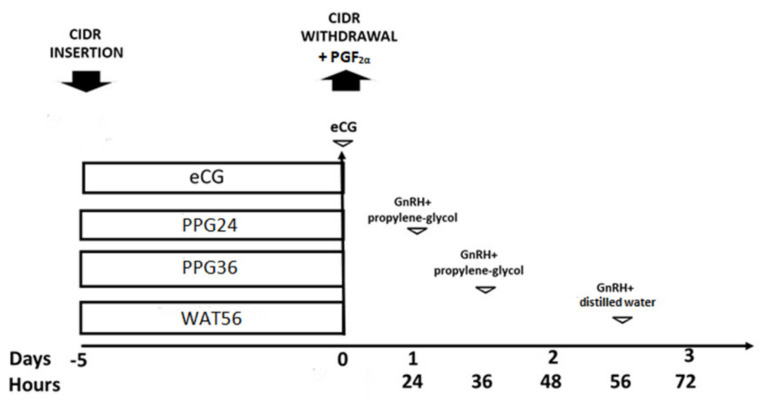
Experimental groups of trial 2, aiming to determine the effect and adequate timing of GnRH administration in either distilled water or propylene glycol on the ovarian response to progesterone-based protocols. All females were treated with one controlled internal drug release (CIDR) device with PGF_2α_ and 400 IU of equine chorionic gonadotrophin (eCG; group eCG, *n* = 8) or a single dose of 50 µg of GnRH in either i.m. distilled water (56 h after CIDR removal; group WAT56, *n* = 8) or s.c. propylene-glycol (24 or 36 h after CIDR removal; groups PPG24, *n* = 11; and PPG36, *n* = 10).

The second trial, developed from the results obtained in the first trial, aimed to determine the effect and adequate timing of GnRH administration in either distilled water or propylene glycol on the ovarian response to progesterone-based protocols for estrus synchronization. Ovarian cyclic activity and ovulation were synchronized, in a total of 37 ewes, by the insertion of one intravaginal progesterone-loaded CIDR (CIDR Ovis, Zoetis, Madrid, Spain) for 5 days with one i.m. injection of 5 mg of PGF_2α_ (Dinolytic, Zoetis, Madrid, Spain) at CIDR withdrawal. Four experimental groups were established, as depicted in Figure 2, being treated with i.m. 400 IU of eCG (Foligon, MSD Animal Health, Madrid, Spain; group eCG, *n* = 8) or with 50 µg of GnRH (Acegon, Lab. Syva, Leon, Spain), as previously described, in either i.m. distilled water at 56 h after CIDR removal (group WAT56, *n* = 8) or s.c. propylene-glycol at 24 or 36h after CIDR removal (groups PPG24, *n* = 11; and PPG36, *n* = 10).

In both experiments, the variables evaluated were follicular dynamics during the follicular phase, percentage of ewes showing estrus signs and ovulation, and ovulation rate. Trial 1 also assessed duration of estrus behavior and plasma estradiol concentrations at estrus onset, whereas trial 2 evaluated timing of ovulation and plasma progesterone concentrations at the following luteal phase.

### 2.3. Occurrence and Timing of Estrus Behavior

Signs of estrus behavior were determined by the use of trained rams in a proportion of one ram/one ewe, twice daily in trial 1 and every 4 h in trial 2, from 24 to 72 h after second PGF_2α_ dose or CIDR removal, respectively. Estrus onset was defined as the time of the first accepted mating, whereas estrus end was defined as time in which the ewes refused to mate. Interval from treatment to estrus onset was determined in both trials, whereas length of estrus was also evaluated in Trial 1. 

### 2.4. Development of Preovulatory Follicles during the Follicular Phase and Timing of Ovulation

The number of all follicles with ≥ 4 mm in size and the diameter of the largest follicles were determined in all the ewes by 7.5 MHz transrectal ultrasonography (Aloka SSD 500, Aloka Co. Ltd., Tokyo, Japan), every 24 h in trial 1 and every 4 h from 24 h after CIDR removal to onset of ovulation in Trial 2. Onset of ovulation was determined by assessing the disappearance of the ovulatory follicles recorded in a previous ultrasonography, as previously described [21].

### 2.5. Plasma Estradiol Concentrations During the Follicular Phase 

Samples of 5 mL of jugular blood were collected concomitantly with estrus detection, from 24 h after second prostaglandin dose or CIDR removal to onset of estrus behavior, with heparinized vacuum blood evacuation tubes (Vacutainer Systems Europe, Becton Dickinson, Meylan Cedex, France). Blood samples were centrifuged at 2000× *g* for 15 min. Thereafter, the plasma was stored at −20 °C until assayed for estradiol-17β determination. Such determination was performed, after sample extraction, by using an enzimoimmunoassay kit (Demeditec Diagnostics GmbH, Kiel-Wellsee, Germany). Sensitivity was 1.4 pg/mL, and intra-assay variation coefficient was 5.7%.

### 2.6. Number and Functionality of Corpora Lutea

Assessment of ovulation and counting of the number of corpora lutea were performed by ultrasonography at day 11 of the induced estrous cycles. The luteal functionality was evaluated in terms of progesterone secretion, by drawing blood samples coincidentally with ultrasound scanning at day 11 and processing them as described above for estradiol. Plasma progesterone concentrations were measured using an enzimoimmunoassay kit (Demeditec Diagnostics GmbH, Kiel-Wellsee, Germany). Sensitivity was 0.045 ng/mL, and intra-assay variation coefficient was 5.4%.

### 2.7. Statistical Analysis

The effects of treatment on the occurrence and onset of estrus behavior and ovulation, ovarian follicle dynamics, number of corpora lutea, and secretion of estradiol and progesterone were assessed by analyses of variance (ANOVA) and chi-squared test by using SPSS 22.0 (IBM Corporation, New York, NY, USA). Statistical analysis of results expressed as percentages was performed after arcsine transformation of the values for each individual percentage, after normality testing of the data. All results in main text and tables are expressed as mean ± S.E.M. and statistical significance was accepted at *p* < 0.05.

## 3. Results

### 3.1. Trial 1: Effect of GnRH Administration in Prostaglandin-Based Protocols

**Figure 3:**
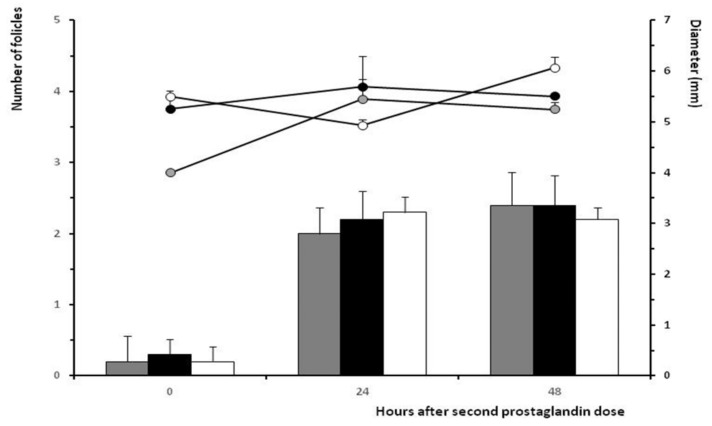
Mean number of ≥ 4 mm follicles (bars) and mean diameter of the largest follicle (lines) from time of second PGF_2α_ to ovulation in ewes treated with two doses of PGF_2α_ and a single dose of distilled water (group CON; white bars and dots) or 50 μg of GnRH in either distilled water (group WAT; grey bars and dots) or propylene-glycol (group PPG; black bars and dots) at 32 h after the second PGF_2α_ dose.

Assessment of the ovarian follicular dynamics showed a similar mean number of ≥ 4 mm follicles and similar mean diameter of the ovulatory follicles at 24 and 48 h after the second prostaglandin F_2α_ dose among treatments (Figure 3), despite such follicles arising from different diameters at 0 h and a faster growth rate per day between 0 and 24 h in the group WAT and between 24 and 48 h in the group CON (*p* < 0.05 for both).

There were no significant differences in the percentage of ewes responding with appearance of estrus and ovulation to the prostaglandin-based treatment in the groups CON and PPG, but such a response was significantly lower in the group WAT (Table 1). Conversely, there were no differences among groups in onset and duration of estrus, plasma estradiol secretion at estrus, and ovulation rate.

**Figure 4:**
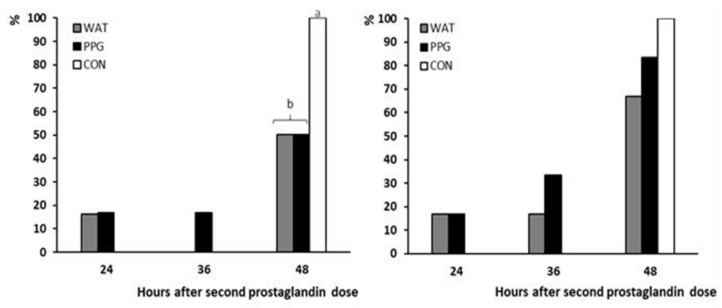
Absolute (left panel) and cumulative (right panel) percentages of sheep showing onset of estrus after treatment with two doses of PGF_2α_ and a single dose of distilled water (group CON; white bars) or 50 μg of GnRH in either distilled water (group WAT; grey bars) or propylene-glycol (group PPG; black bars) at 32 h after the second PGF_2α_ dose. Different superscripts indicate significant differences among treatments (a ≠ b: *p* < 0.05).

The distribution of occurrence of estrus over time showed that, although some of the animals were in estrus prior to GnRH application, almost all of the responding animals showed the onset of estrus behavior between 36 and 48 h after the second prostaglandin dose, with a higher percentage of them in the group CON (*p* < 0.05; Figure 4).

### 3.2. Trial 2: Efficiency and Timing of GnRH Administration in Progesterone-Based Protocols

Occurrence of estrus behavior was similar among groups eCG, WAT56, and PPG36 (87.5%, 80%, and 100% of the ewes, respectively) and lower in the group PPG24 (18.2%; *p* < 0.05). However, all ewes showed ovulations with similar ovulation rate among groups but progesterone concentrations higher in groups eCG, WAT56, and PPG36 than in the group PPG24 (*p* < 0.05; Table 2).

**Figure 5:**
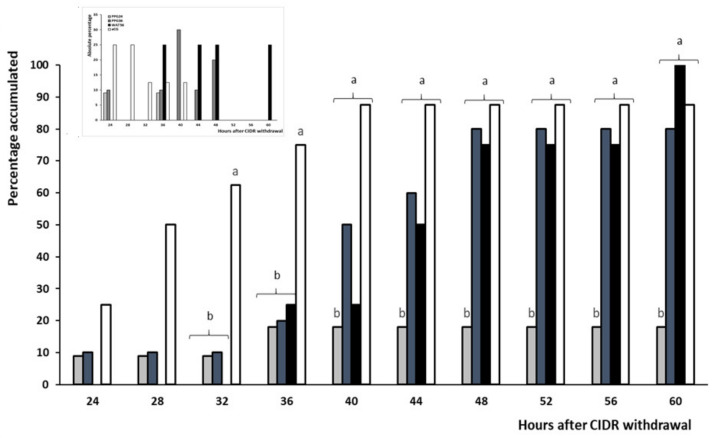
Timing of onset of estrus in ewes treated with one CIDR with PGF_2α_ and 400 IU of eCG (at CIDR removal; group eCG, white bars) or 50 µg of GnRH in either distilled water (56 h after CIDR removal; group WAT56, black bars) or propylene-glycol (24 or 36 h after CIDR removal; groups PPG24 and PPG36, with light and dark grey bars, respectively). Cumulative percentage of animals is represented in the main panel, whereas absolute percentages in each interlude are represented in the inset graph. Different letters indicate statistical differences between groups (a ≠ b: *p* < 0.05).

The onset of estrus behavior (Figure 5) occurred significantly earlier in groups eCG and PPG24 (around 30 h for both) than in groups PPG36 and WAT56 (around 40 and 47 h, respectively; *p* < 0.05). 

**Figure 6:**
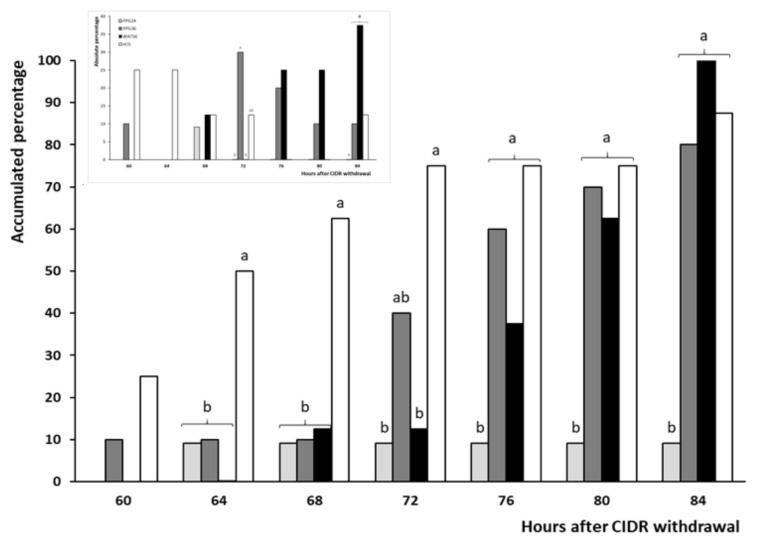
Timing of onset of ovulation in ewes treated with one CIDR with PGF_2α_ and 400 IU of eCG (at CIDR removal; group eCG, white bars) or 50 µg of GnRH in either distilled water (56 h after CIDR removal; group WAT56, black bars) or propylene-glycol (24 or 36 h after CIDR removal; groups PPG24 and PPG36, with light and dark grey bars, respectively). Cumulative percentage of animals is represented in the main panel, whereas absolute percentages in each interlude are represented in the inset graph. Different letters indicate statistical differences between groups (a ≠ b: *p* < 0.05).

Afterwards, as depicted in Figure 6 and Table 2, occurrence of ovulation was also earlier in groups eCG and PPG24 (around 66–68 h) than in the group WAT56 (around 80 h, *p* < 0.05), with intermediate values for PPG36 (around 75 h). However, such differences were mostly related to the earlier appearance of estrus, as the interval estrus-ovulation was similar among groups (Table 2). 

**Figure 7:**
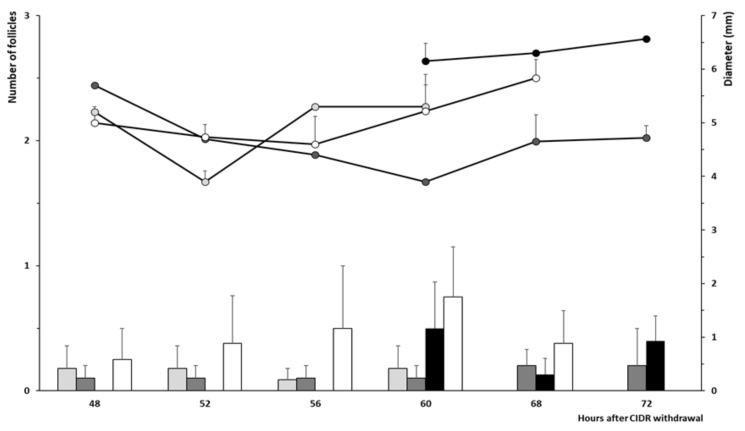
Mean number of ≥ 4 mm follicles (bars) and mean diameter of the largest follicle (lines) from 48 h after CIDR withdrawal to ovulation in ewes treated with 400 IU of eCG (at CIDR removal; group eCG, white bars and dots) or 50 µg of GnRH in either distilled water (56 h after CIDR removal; group WAT56, black bars and dots) or propylene-glycol (24 or 36 h after CIDR removal; groups PPG24 and PPG36 with light grey and dark grey bars and dots, respectively).

The assessment of follicle dynamics from 48 h after CIDR withdrawal to the occurrence of ovulation (Figure 7) showed similar patterns among groups, except for a larger diameter of the largest follicle in the group WAT56 than in the other groups (*p* < 0.05). 

## 4. Discussion

The results from the present study confirm previous data indicating that the ovarian response to the administration of GnRH is highly dependent on the timing of such administration and may indicate a similar situation when using propylene-glycol as a cosolvent. Previous studies have shown that GnRH must be applied at least 24–36 h after progestagen removal or luteolysis [22]. Its earlier application indeed induces an early LH surge, which may block the ovarian steroidogenesis and cause anovulation and luteinization of the preovulatory follicle [23]. There are other studies that indicate that even GnRH administration at 24 or 30 h after induction of luteolysis with PGF_2α_ may compromise reproductive performance [23,24,25]. These results are supported by our current data. First, in the GnRH-treated group, the largest follicle was in the static phase of growth around estrus, whilst such a follicle was growing in the control group. Concomitantly, although differences did not reach statistical significance, possibly due to the low number of animals in each group, plasma estradiol concentrations at estrus onset in the ewes injected with GnRH both in distilled water and PPG at 32 h after PGF_2α_ administration were lower than in the controls. Finally, one-third of ewes from the WAT group did not show the occurrence of estrus, and half of them did not ovulate. From these results, administration of GnRH earlier than 36 h would not be useful for inducing a better synchronization of ovulation in PGF_2α_-based treatments, in agreement with data from Olivera-Muzante and co-workers [25].

The results found in the first trial when assessing follicle dynamics and estradiol secretion in ewes treated with GnRH in propylene-glycol were similar to sheep treated with GnRH in distilled water. However, around 83% of these sheep showed estrus signs, and all of them ovulated, which supports the idea that administration of GnRH in a long-acting cosolvent like propylene-glycol via subcutaneous would induce better results than in the case of intramuscular saline solution or distilled water. In any case, the objective of this first trial was to determine if an early administration of GnRH in propylene-glycol would also block the appearance of estrus and ovulation or would be useful for inclusion in progesterone-based treatments. From the positive results obtained in the first trial, we developed the second experiment.

The results found in the second trial confirmed that an early administration of GnRH following the induced luteolysis alters the final growth of the ovulatory follicles. In the group treated with GnRH at 24 h after CIDR withdrawal (group PPG24), estrus behavior was only observed in two of the 11 sheep; one of them was already in estrus at the GnRH injection and ovulated, whereas the other ewe developed an anovulatory follicle. Afterwards, all of the ewes in this group were found to be ovulated at the ultrasonographic screening performed 11 days after CIDR removal, but showed lower plasma progesterone concentrations than the remaining groups. Moreover, progesterone concentrations in the subsequent luteal phase were lower in PPG24 compared to the other groups. It is well known that the occurrence of an inadequate follicular development leads to subnormal corpus luteum formation, causing low levels of secretion of progesterone [26]. Secretion of estradiol during preovulatory stages and secretion of progesterone during the first days of the luteal phase of sheep play an important role on the expression of several hormones and signaling factors in the oviduct and uterine secretions, crucial for early embryo development [27,28,29]. Therefore, according to the results of the present study, the conditions created by the early GnRH administration would not promote adequate conditions for the final follicular growth and the conception in the ewe [8,30]. 

Conversely, the results obtained with the use of GnRH at 36 h after CIDR withdrawal (group PPG36) are comparable to the control groups eCG and WAT56 and are therefore very promising. In fact, 80% of the animals in the group PPG36 developed estrus in a narrow timing (75% between 36 and 48 h after CIDR removal), and all of them ovulated, also in a narrow window (87.5% between 72 and 84 h after CIDR removal, with 62.5% between 72 and 76 h). The screening of corpora lutea also showed similar ovulation rate and plasma progesterone concentrations to the control groups. The main difference among the groups eCG, PPG36, and WAT56, in agreement with previous studies [8], were mainly determined by the timing of onset of estrus behavior (i.e., the time needed by the preovulatory follicle to reach its maximal estradiol secretion and therefore to induce estrus signs), as the intervals between the preovulatory LH surge and the ovulation were similar among the three treatments. In fact, timing of ovulation in the group PPG36 was intermediate between groups eCG and WAT56, which, besides the narrow range of appearance of estrus and ovulation, indicated a good synchronization of the terminal growth phase of ovulatory follicles. 

These results suggest that administration of GnRH in propylene-glycol at 36 h after CIDR withdrawal may constitute a good alternative for protocols based on FTAI during the breeding season. However, such a hypothesis needs to be tested under field conditions because the design of the present study was mainly focused on studying the characteristics of follicle dynamics, estrus, and ovulation (which require a high number of successive samples from a small number of animals), rather than the fertility yields (which require a large number of animals). Concomitantly, the current results showing that administration of GnRH in propylene-glycol at 36 h after CIDR withdrawal is useful to stimulate the adequate development and maturation of preovulatory follicle may set the basis for its application during non-breeding season, when eCG shortage may be critical because gonadotrophin secretion and therefore ovulation are depressed [31].

## 5. Conclusions

Protocols based on a short-term CIDR treatment and a single dose of GnRH in propylene-glycol at 36 h after CIDR removal may constitute an alternative to traditional protocols based on the administration of a single dose of eCG at CIDR removal during breeding season. Further research for assessing its implementation and usefulness during non-breeding season needs to be undertaken.

## Figures and Tables

**Table 1:** Percentage and timing of occurrence of estrus behavior, estradiol concentration at estrus, and occurrence and number of ovulations in ewes treated with two doses PGF_2α_ and a single dose of distilled water (CON) or 50 μg of GnRH in either i.m. distilled water (WAT) or s.c. propylene-glycol (PPG) at 32 h after the second PGF_2α_ dose.

Group	PPG	WAT	CON
Occurrence of estrus (%)	83.3 (5/6) ^a^	66.7 (4/6) ^b^	100 (6/6) ^a^
Onset of estrus (h)	40.8 ± 4.3	42.0 ± 4.8	48.0 ± 0.1
Length of estrus (h)	32.4 ± 8.0	27.0 ± 4.6	38.0 ± 4.8
Plasma estradiol at estrus (pg/mL)	3.0 ± 1.6	2.9 ± 0.2	5.5 ± 1.2
Occurrence of ovulation (%)	100 (6/6) ^a^	50 (3/6) ^b^	100 (6/6) ^a^
Ovulation rate (n)	1.6 ± 0.3	1.3 ± 0.2	1.5 ± 0.2

Different superscripts indicate significant differences among treatments (a ≠ b: *p* < 0.05).

**Table 2:** Percentage, timing of occurrence and intervals of estrus behavior and ovulation, number of ovulations, and plasma progesterone concentrations in ewes treated with CIDR with PGF_2α_ and 400 IU of eCG (at CIDR removal; eCG) or 50 µg of GnRH in either i.m. distilled water (56 h after CIDR removal; WAT56) or s.c. propylene-glycol (24 or 36 h after CIDR removal; PPG24 and PPG36).

Group	PPG24	PPG36	WAT56	eCG
Occurrence of estrus (%)	18.2 (2/11) ^b^	80.0 (8/10) ^a^	100 (8/8) ^a^	87.5 (7/8) ^a^
Timing of estrus (h)	30 ± 2.5 ^a^	40 ± 2.4 ^b^	47 ± 3.2 ^b^	30.1 ± 1.5 ^a^
Occurrence of ovulation (%)	100 (11/11)	100 (10/10)	100 (8/8)	100 (8/8)
Timing of ovulation (h)	68 ^a^	74.5 ± 2.3 ^a,b^	79.5 ± 2.2 ^b^	66.3 ± 3.8 ^a^
Interval estrus-ovulation (h)	40	34 ± 1.3	32.5 ± 1.9	36.1 ± 2.5
Ovulation rate (n)	1.7 ± 0.2	1.8 ± 0.2	1.6 ± 0.1	1.5 ± 0.2
Plasma progesterone (ng/mL)	4.5 ± 0.4 ^a^	6.1 ± 0.6 ^b^	5.1 ± 0.6 ^b^	5.3 ± 1.3 ^b^

Different superscripts indicate significant differences among treatments (a ≠ b: *p* < 0.05).
